# A primate grammar enabling incremental processing

**DOI:** 10.1016/j.isci.2025.112229

**Published:** 2025-03-20

**Authors:** Quentin Gallot, Yves Tillé, Cassandre Depriester, Steven Moran, Klaus Zuberbühler

**Affiliations:** 1 Institute of Biology, University of Neuchâtel, Neuchâtel, Switzerland; 2 Taï Monkey Project, Centre Suisse de Recherches Scientifiques en Côte d'Ivoire, Ivory Coast, Côte d’Ivoire; 3 Institute of Statistics, University of Neuchâtel, Neuchâtel, Switzerland; 4 ENES Bioacoustics Research Laboratory, CRNL, University of Saint-Etienne, Saint-Etienne, France; 5 Department of Anthropology, University of Miami, Coral Gables, FL, USA; 6 Linguistic Research Infrastructure, University of Zurich, Zurich, Switzerland; 7 School of Psychology and Neuroscience, University of St Andrews, St. Andrews, UK

**Keywords:** Biological Sciences, Zoology, Evolutionary biology

## Abstract

Characterizing the structure and function of animal communication systems provides insights into the cognitive and evolutionary processes shaping signal complexity. One key question is whether and how call sequences allow potential listeners to make predictions about the call-eliciting referents. Here, we investigated whether primate call sequences contained properties that enabled such predictive processing. We analyzed several years of experimentally elicited alarm responses from a West African forest primate, wild olive colobus monkeys. Using Kullback-Leibler divergence and prediction gain approaches, we identified a simple primate grammar that allowed predictions of referents from only minimal input. In particular, sequence-initial positions reliably discriminated urgent from non-urgent threats while the following positions increased the referential specificity regarding two main predators (eagles and leopards) and non-predatory disturbances (falling tree parts). Sequences often contained further calls, which may allow callers to either confirm the referent or to alter the conveyed information. We concluded that animal communication can contain features adapted for predictive, incremental processing, suggesting evolutionary roots older than language.

## Highlights

• Olive colobus monkeys have a minimal vocal repertoire with only two calls
• Calls are produced in context-specific sequences that adhere to a simple grammar
• Initial calls state urgency, later calls refine referents and allow eventual corrections
• Information gain and entropy reduction is incremental, allowing predictive processing

## Graphical abstract

**Figure:**
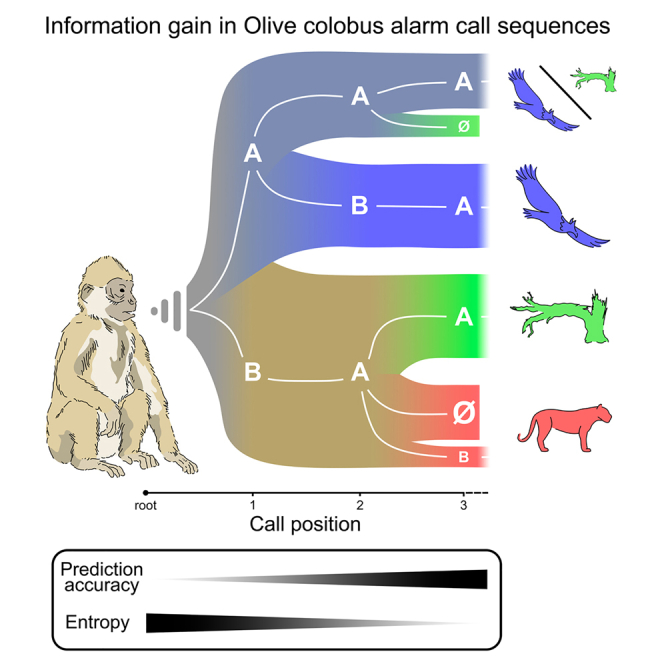

## Introduction

Grammar can be defined as a system of rules that specify how signal sequences are formed, combined, and used to generate meaning.^1^ While the grammar of animal communication systems is nowhere near as intricate as the grammar of human languages, there are now several examples of how animals combine signals along different principles, although this does not always have a demonstrated impact on generating meaning.^2^ For example, Girard-Buttoz et al.^3^ showed that chimpanzees (*Pan troglodytes*) produce long and highly flexible vocal sequences of up to 10 call types with ordering and recombination properties, but the relation of the different structures to external events has remained largely unclear. Another puzzle concerns the phylogeny of call sequences with unexplained interspecies differences in complexity, such as gibbon song that functions in intergroup communication, but varies considerably in complexity across and even within species.^4^ For example, agile gibbons (*Hylobates agilis*) frequently produce highly complex songs from 13 different notes,^4^ whereas lar gibbons (*Hylobates lar*) produce songs with an average of only 6 notes.^5^^,^^6^

Despite well-documented structural richness, there is not much theory available to explain the communicative function and evolutionary reasons for structural diversification in animal call sequences, apart from a few standard hypotheses. First, it is always possible that the information content has been underestimated. In particular, the use of support vector machine techniques tends to reveal additional layers of information conveyed at more subtle levels.^7^ For example, analysis of wild chimpanzee ‘pant-hoot’ utterances revealed highly complex call sequences consisting of four phases, each conveying different aspects of reality in the information flow, such as caller identity, age, social status and general context.^7^ Second, signalers occasionally produce complex sequences due to a need to address different audiences with the same sequence. For example, chimpanzees that have become victims of aggression often produce call sequences consisting of both ‘waa’ barks and screams, with only the latter being influenced by audience composition. Screams appear to be delivered strategically as attempts to enlist the help of bystanders,^8^ whereas ‘waa’ barks are directed at the aggressors to signal motivation for retaliation.^9^ Third, signal sequences often have built-in redundancies, presumably to improve signal transmission and reception in difficult environments both in terms of sound transmission and interference with other species’ vocal behavior.^10^ Finally, complexity can emerge, not as part of a ‘grand plan’, but as by-products of signalers continuously commenting on ongoing changing events and social interactions, with signal structures tracking relevant changes hereby updating nearby audiences on relevant events.^8^^,^^11^^,^^12^

Alarm contexts have been especially fruitful in studying the evolution of signal sequences. Here, it has been argued that scattering information over long sequences is unlikely to be adaptive, especially if one primary function of alarm signals is to enable genetically or socially valuable receivers to make survival decisions rapidly and accurately.^13^ The fact that many species nevertheless produce exceedingly long alarm call sequences therefore requires additional explanations and is indicative of additional functions. In some cases, complexity itself can function to refer to external events, for instance, by increasing the number of elements in relation to the immediacy of the danger.^13^ Oftentimes, the same predator can exert different types of threat due to its hunting behavior (e.g., perched, circling or attacking eagles), location (above vs. below), distance (close vs. far) or visibility (hidden vs. visible) (e.g., Griesser^14^), suggesting that communication systems should evolve means to convey such information. Finally, as predicted by the previous hypotheses, predation events are usually complex and lengthy interactions, requiring continuous adjustments that allow callers to respond to relevant changes in their assessment of such ongoing events.

Whatever additional factors favor complex signal sequences, predation attempts are highly dangerous and require immediate responses, suggesting that selection should favor communication systems that are capable of transmitting the most urgent information first.^15^^,^^16^ For example, eagles will mostly attack monkeys by surprise and at short distances, requiring rapid flight responses into dense cover, a serious constraint for many animal species hunted by raptors.^17^ For example, New Holland honeyeaters (*Phylidonyris novaehollandiae*) produce long, multi-note alarm signals to aerial threats, but the danger-specific acoustic variants are already available in the first note.^13^ Another example is black-fronted titi monkeys (*Callicebus nigrifrons*), a species also producing long call sequences, with callers consistently referring to the most urgent predator information first, before less urgent social information,^18^ in compliance with what has been determined as the Urgency Principle.^15^^,^^16^ However, there are also other examples. Research comparing three populations of putty-nosed monkeys in Ivory Coast, Nigeria and the Congo Republic found that males differed in how they assembled call sequences to the same referents. Notably, callers from the Congo Republic commenced alarm call sequences with a series of ‘pyows’, regardless of the referent, most likely because ‘pyows’ are rich in individual identity.^19^ Subsequently, males either produced series of ‘hack’ alarms to crowned eagles, their most dangerous predator, or further ‘pyows’ to terrestrial dangers.^20^ In the other two populations, male responses to eagles always started with ‘hacks’. Finally, in the Nigerian population only, if ‘hacks’ were preceded by ‘pyows’, they took on a new meaning with the function of rallying others for group movement.^20^^,^^21^

Here, we investigated the vocal system of a highly cryptic, forest-living African monkey with promise to further advance theories of signal complexification, the alarm call behavior of olive colobus monkeys (*Procolobus verus*). Both males and females produce vocalizations as part of a simple repertoire consisting of two call types^22^: low-pitched, short ‘A’ and high-pitched, long ‘B’ calls. Both calls travel well through dense forest,^22^^,^^23^^,^^24^^,^^25^^,^^26^ and can be heard beyond the caller’s own home range. Crucially, the calls are always part of longer structured sequences,^22^ up to about two dozen calls, given mainly in response to disturbances, which includes the main predators, leopards and crowned eagles.^22^^,^^23^^,^^24^

In a previous study, we have identified four main sequence patterns,^22^ i.e., repetitions of ‘A’ calls, ‘BA-gram(s)’, ‘A’ call(s) followed by ‘BA-gram(s)’, and ‘BA-gram(s)’ followed by ‘A’ call(s). ‘BA-grams’ were found in the main body of the sequence. If preceded by ‘A’ call(s) the caller referred to a crowned eagle; if succeeded by ‘A’ call(s), the caller referred to a falling tree. In the absence of such ‘A’ affixes, however, the caller referred to a leopard, all being constant between olive colobus groups and with no visible effect on whether the danger was on the ground or a tree^22^ (see Figures S1–S3 for spectrographic representations; Data S1 for the corresponding recordings).

Based on other primate studies, we hypothesized that the composition of olive colobus call sequences was context-dependent with information about the predator type conveyed early in the sequence, and with later parts serving additional functions, such as ensuring that the information is perceived through redundancy or by referring to other aspects of the ongoing event.

Predation attempts are usually messy, chaotic and confusing affairs. Attacks can happen very suddenly, unexpectedly and rapidly so that callers routinely operate under time pressure and with incomplete information, unable to carefully produce a referentially unambiguous alarm call response. Forced to instantly react, they are usually relegated to take partially informed guesses about the actual cause of the disturbance, and their vocal behavior will reflect this fact.^27^ Although it may not be possible for callers to carefully plan and produce referentially concise utterances during the early phases of a predation attempt, they can still produce valuable information early on, especially if subsequently followed by more precise information.

Something similar appears to take place in human languages, where incremental processing is the default mechanism at multiple linguistic levels. For example, listeners predict the next word in a sentence based on context and grammatical cues (e.g.,^28^^,^^29^). When processing speech, phonetic cues are integrated over time; for instance, the initial sounds of words can signal likely completions, such as/bei/leading to ‘baby’ (e.g.,^29^^,^^30^^,^^31^^,^^32^). Moreover, speech sounds at the beginning of words tend to be more informative and disambiguatory than later sounds. For example, in English, the word-initial consonants in ‘best’ and ‘pest’ provide more crucial information for recognition than the shared vowel sound (e.g.,^33^^,^^34^). These properties reflect the brain’s capacity for efficient and predictive linguistic processing. Given that all spoken languages operate by incremental disambiguation, both in production and comprehension,^28^^,^^29^ and given how the brain is thought to operate more generally by constantly generating and updating mental models of the environment with predictive coding,^35^^,^^36^^,^^37^^,^^38^ we expected to find some form of evolutionary continuity, especially in cognition but possibly also in communication.

To investigate this hypothesis, we analyzed a dataset of *N* = 284 experimentally elicited call sequences spanning over nearly a decade of research recorded from over 30 different, highly elusive and highly cryptic olive colobus groups in Taï National Park, Ivory Coast (period 1: *N* = 11 different groups, KZ, 1994–1999; period 2: *N* = 18 different groups, QG, 2021–2022; CD, 2022; Table 1). This forest-living African primate forms small but highly fluid multi-male, multi-female social groups of 2–15 individuals,^39^ but otherwise engages in very little social behavior.^40^ The olive colobus social system is also very unusual, with individuals occasionally visiting neighboring groups, with commutes taking place when their host groups, mostly Diana monkeys, are near each other.^41^ In alarm situations, both males and females can vocalize, but this happens only rarely and with a most basic repertoire of two main call types.^22^

**Table 1:** Olive colobus dataset composition

Dataset	Description	Experimenter	N trials with response	Total N trials	Year	N groups^a^
1	Predator playbacks	KZ	18	24	1994–99	11
2	Predator and tree playbacks	QG	70	157	2021–22	16
	Tree playbacks	CD	21	44	2022	15
3	Predator models	QG	10	25	2022	8

a Based on the spatial estimates.

Using Kullback-Leibler divergence,^42^^,^^43^ a concept from information theory that measures the dissimilarity between two probability distributions, we tracked the information content of the utterances produced by a large number of individuals responding to experimentally presented, evolutionarily relevant events to investigate whether and how these call sequences contain a grammar to allow prediction of referents from only minimal input.

## Results

### Sequence structure

We first sought to characterize the structure of the various sequences (*N* = 284) recorded between 1994 and 2022, which resulted in three different datasets (Table 1), with *N* = 119 trials overall consisting of systematically elicited vocal responses to different types of dangers. Calls from datasets 1 and 2 were in response to playbacks of predator vocalizations (leopard growls and eagle shrieks) and sounds of falling trees, while data from dataset 3 consisted of calls recorded in response to visual models of a leopard (*N* = 119 trials with vocal responses; see STAR Methods for details on dataset 1: *N* = 18 trials; dataset 2: *N* = 91 trials; dataset 3: *N* = 10; Table 1).

**Figure 1:**
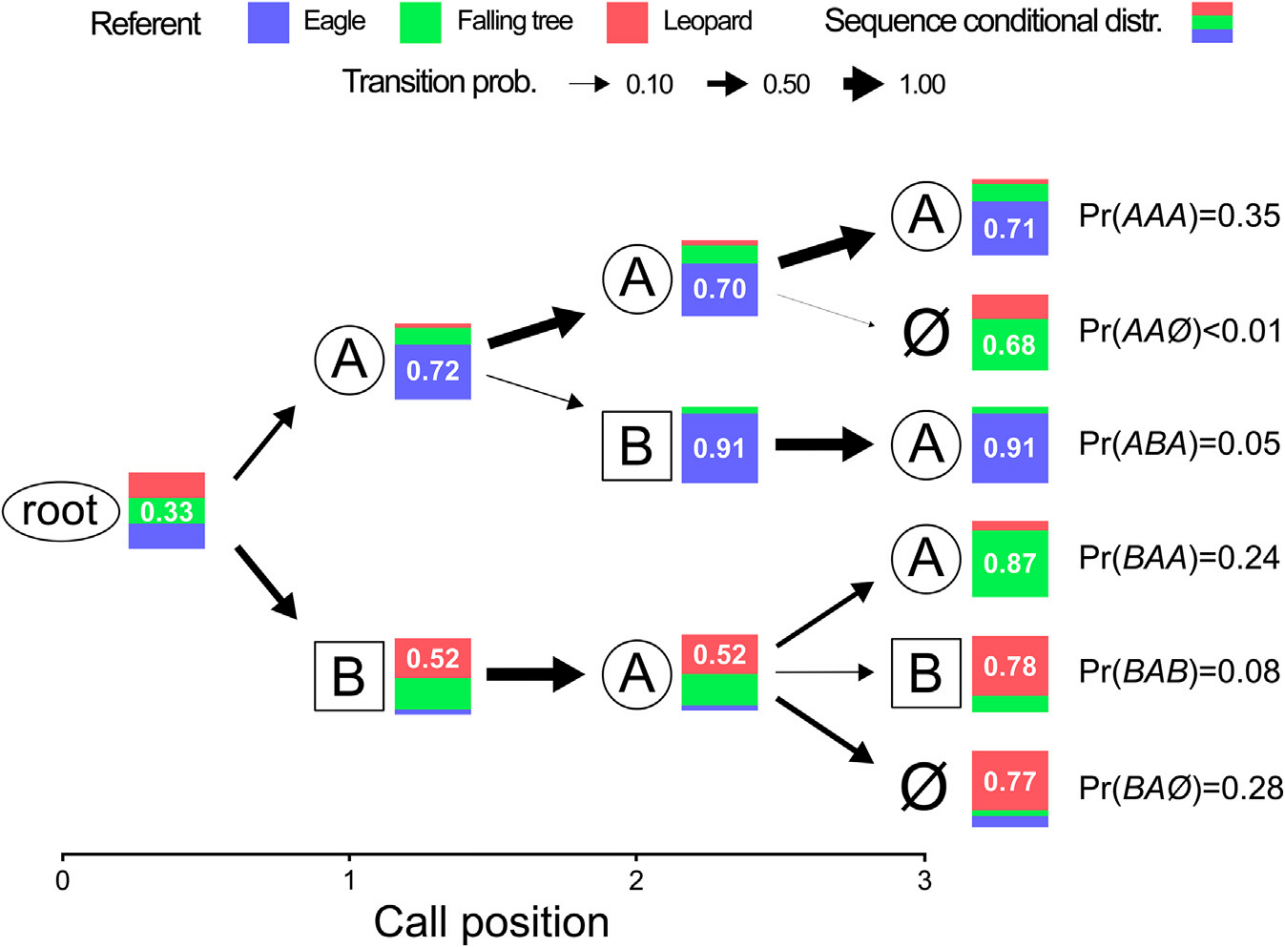
Trie representation of the three first positions in olive colobus alarm sequences Each node represents a call or a position (circle = low-frequency short ‘A’ call type, square = high-frequency long ‘B’ call type, ‘root’ = start, ‘∅’ = end). The x-coordinates represent the position of the call in the sequence, the arrow direction represents the order of combination, and its thickness the transition probability. Each node is associated with a bar plot representing the weighted probability of the nature of the disturbance conditional to the sequence produced at the corresponding position. Each color corresponds to a possible danger (blue = eagle, green = falling tree, red = leopard). The value inside each bar plot refers to the maximum probability, which is also the probability of accuracy of the nature of the disturbance.

**Figure 2:**
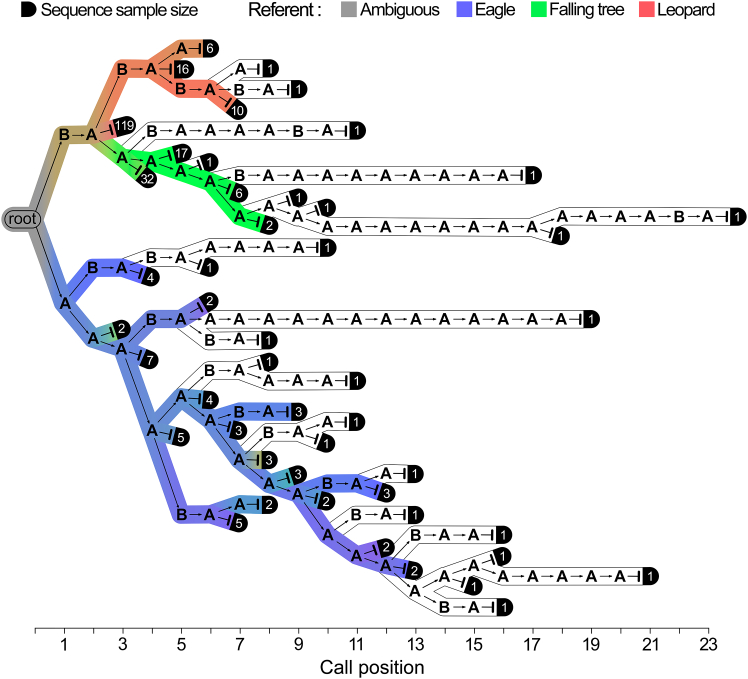
Trie representation of *N* = 284 call sequences recorded in response to 3 different danger events: leopard, eagle, and falling tree Each node represents a call or a position. The x-coordinates represents the position of the call in the sequence, the arrow direction represented the order of combination, and a sequence ends when a node is followed by ‘⊣’. The number at the end of each branch represents the sample size of the corresponding sequence in all datasets. Only sequences that have been recorded more than once are colored (white background for sequences with *N* = 1). Nodes are colored in the background, and a transition between two nodes is represented as a color gradient. Each RGB color vector (0 < R < 255, 0 < G < 255, 0 < B < 255) corresponds to the sequence conditional distribution to each call-eliciting event with red (255,0,0) = leopard, green (0,255,0) = falling tree, and blue (0,0,255) = eagle (*N*_eagle_ = 39, *N*_falling tree_ = 79, *N*_leopard_ = 166). In consequence, the purer the color, the more danger-specific the sequence is. An ambiguous signal here is gray (153,153,153). Audio recordings and spectrographic representations of call sequences are available in Data S1 and Figure S1–S3.

For all datasets, we compiled tries (Figures 1 and 2), with nodes corresponding to individual call positions (circle = ‘A’, square = ‘B’, ‘root’ = start, ‘∅’ = end). We then calculated the transition probabilities between the nodes and the probabilities of sequences to be produced conditional to the nature of the disturbance (Figure 1; Table S1). A sequence ended if a node was followed by ‘⊣’ (Figure 2), which corresponded to >1s of silence.

### Prediction accuracy

We found that the first position disambiguated between potential eagle and non-eagle dangers, arguably the most urgent decision to be taken during a predation attempt (Figure 1). Sequences initiated by a ‘B’ call excluded an immediate danger (presence of an eagle) and predicted the disturbance to be caused by a leopard with 52% accuracy. Sequences initiated by an ‘A’ call predicted an eagle as the source of disturbance with 72% accuracy, while retaining a 22% probability of being associated with a falling tree. For ‘A’-initiated sequences, the second position allowed the caller to either maintain the state of unspecified urgency (by producing another ‘A’) or to produce a ‘B’ call, which increased the accuracy of predicting an eagle to 91% (Figure 1). The third position, then, further specified the referent for both ‘B’- and ‘A’-initiated sequences: ‘BA∅’ referred to leopard-caused disturbances, ‘BAA’ to falling tree-caused disturbances, and ‘AAA’ to urgent unspecified danger, most likely an eagle (Figure 1). The modality of how callers detected the predators had no influence on sequence structure (i.e., visual or acoustic leopard models, Figure S4). Similarly, the location of the disturbance was equally irrelevant (i.e., from the ground or from within the trees, Figure S4).

### Information gain

**Figure 3:**
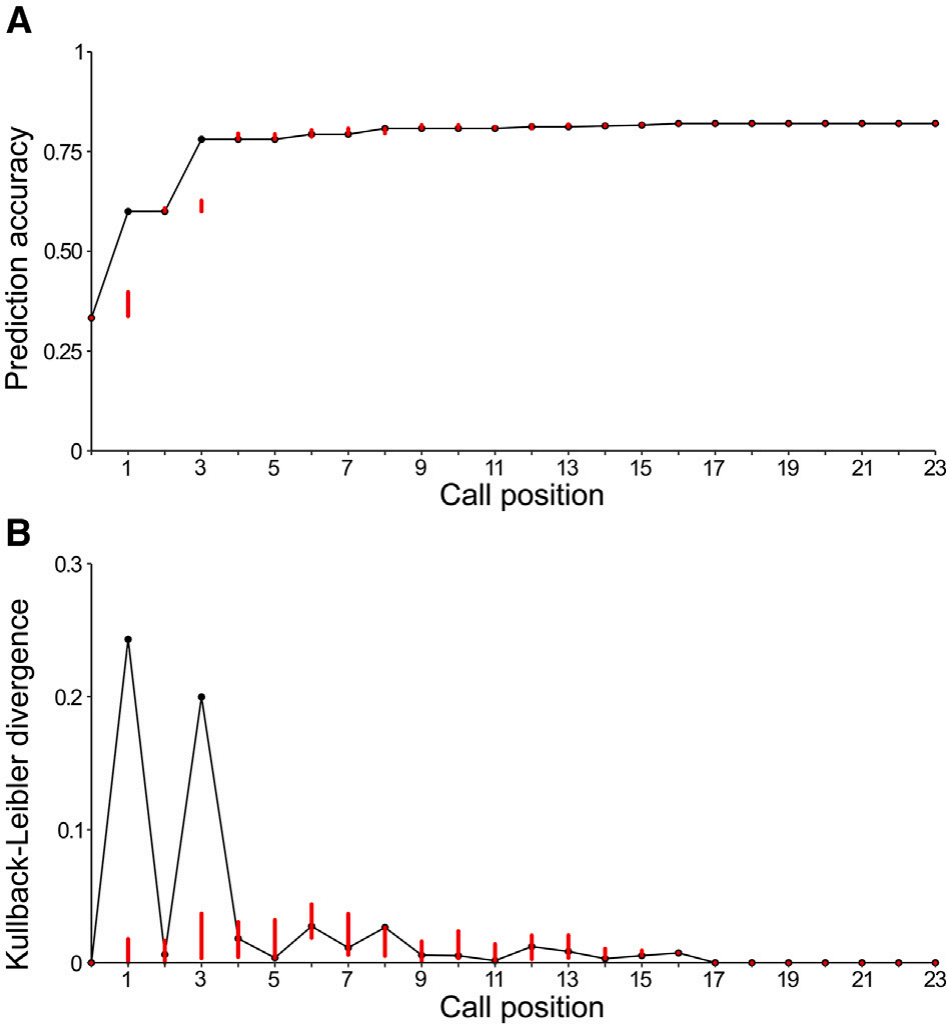
Information gain in olive colobus sequence production Variation of (A) prediction accuracy and (B) Kullback-Leibler divergence as a function of the number of calls produced. Higher Kullback-Leibler divergence indicates greater information gain; divergence was normalized to lie between 0 and 1, using a base-3 logarithm. For each call position, the red segment corresponds to a confidence interval calculated using *N* = 3,000,000 permutations, under the hypothesis that the call position in step t does not provide any additional information to that already available in step t-1. These confidence intervals were constructed by randomizing the stimuli within the call categories defined at step t-1. If the index value at a call position is not within the interval, this means that the call position at step t provides significant additional information to that already known at step t-1.

To further investigate whether olive colobus call sequences allowed recipients to make early predictions from incomplete input, we analyzed the information gain along each sequence (*N* = 284) and compared the index values to chance level (i.e., a confidence interval at step t constructed by randomizing the stimuli within the call categories defined at step t-1, *N* = 3,000,000 permutations; Figure 3; Table S2 and S3). We first calculated the ‘Prediction Gain’ relative to the actual disturbance by tracking the changes in prediction accuracy along the call positions of the sequences (confusion matrix, Figure 3A). For example, if no call had been uttered, then each of the three possible disturbances had a weighted probability of 1/3 (see STAR Methods). Any subsequent increase in prediction accuracy for later call positions would indicate a gain in information regarding the nature of the disturbance.

The second information theory approach involved calculating Shannon entropy,^44^ i.e., the uncertainty in a set of possible outcomes, which is a standard way to quantify the information content of a system (Figure 3B). Before any calls were produced, uncertainty was set to a maximum of 1, whereas each disturbance again had a probability of 1/3. We then quantified the change in entropy as sequence production progressed using Kullback-Leibler Divergence,^42^^,^^43^ which measures the information gain between successive call positions regarding the nature of the disturbance.

We found that information gain about the nature of the disturbance (i.e., eagle, leopard, falling tree) occurred significantly only at the first and third positions of the sequence, with a Kullback-Leibler divergence being close to 0 and within chance level for the other positions in the sequence (Figure 3A; Table S2). The variation in probability of accuracy across call positions supported this hypothesis, with an important Prediction Gain about the nature of the disturbance being only significant at the first and third positions of the sequence. A plateau phase started at the third position with a maximum prediction accuracy of 82%.

## Discussion

Olive colobus monkeys have an unusually simple vocal repertoire, compared to what is normally reported for non-human primates, consisting of only two basic call types.^22^ These calls are combined in sequences following a simple grammar that potentially allows recipients to make increasingly accurate predictions about the referent—eagles, leopards, or collapsing trees^22^—three distinct events that pose unique threats and differ in urgency by which individuals need to react.^17^^,^^45^^,^^46^^,^^47^ Falling trees or breaking branches can pose a mortal danger, especially if large trees bring down adjacent smaller trees, but the threat is not directly targeted at a specific individual and often manageable with evasive action. Crowned eagles and leopards, on the other hand, are highly dangerous and persistent primate predators,^48^^,^^49^ but differ in the type of danger they pose. Leopards hunt by hiding near monkey groups to attack individuals that come to the ground.^45^^,^^46^^,^^50^ Once detected, they are much less dangerous, since monkeys have a locomotor advantage within the continuous forest canopy. Eagles, on the other hand, can attack from almost anywhere and at very short distances, causing a permanent, ubiquitous, and grave security risk for most primates.^17^

By analyzing a large dataset of experimentally induced vocal responses collected over four decades of research, we found that olive colobus alarm sequences possessed a simple grammar by which signalers first referred to urgency, with the initial call position distinguishing between events requiring immediate action (‘A’ = eagle) and events not requiring immediate action (‘B’ = leopard or falling tree).

The second call position then did not provide further information, neither for ‘A’- nor ‘B’-initial sequences, but crucially allowed callers to make eventual corrections of the referent. This was not relevant for B-initial sequences, which were always followed by an ‘A’ call, and referred to dangers that did not require instant locomotor responses. Sequence starting with ‘B’ were always followed by ‘A’, hereby not providing further information, as ‘BA’ continued to refer to both leopards and trees (Figure 1). For ‘A’-initiated sequences (typically eventually referring to an eagle), however, callers had the option to either produce a ‘B’ call, in which case the eagle was confirmed, or to remain uncertain by producing further ‘A’ calls. This appears to be highly adaptive because eagles often hide within the forest canopy and can produce sounds and movements that resemble the onset of a collapsing tree or breaking large branch. By initiating a sequence with an ‘A’, callers can invoke the possibility of eagle presence, which they can then either confirm (by producing a ‘B’ call) or not confirm (by producing one or more further ‘A’ calls). As soon as a ‘B’ call is produced, however, the eagle referent appears to be confirmed.

Further disambiguation of the referent then occurred from the third position onward, regardless of the overall length of the sequence. Here, if the caller produced an ‘A’ in third position after a ‘BA-gram’, it referred to a falling tree. In response to falling trees, callers almost always continued with ‘A’ calls, suggesting that ‘BAA’ functioned as the primary reference for tree. In response to leopard danger, the most common outcome was termination of the sequence after ‘BA’, so that the third position remained empty. If callers continued to vocalize, then this was often by producing at least one more ‘BA-gram’, suggesting that ‘BA’ function was the primary reference for leopard. In line with the observed patterns, we found that increases of Kullback-Leibler divergence and prediction accuracy were only visible at the first and third positions along the utterances. Therefore, the information gain about the nature of the disturbance (i.e., eagle, leopard, or tree) occurred early in the sequence, allowing recipients to make early and reliable predictions from incomplete input.

Finally, we checked whether sequences differed depending on whether callers saw or heard the predator (e.g., Arnold et al.^51^) or whether the predator was in the tree or on the ground (e.g., Zuberbühler et al., Cäsar et al., and Berthet et al.^52^^,^^53^^,^^54^), but found no such evidence.

In the past few years, a striking parallel has appeared between how linguistic meaning is computed by the brain and how neural networks generate linguistic material in text creation, translation and completion.^55^^,^^56^^,^^57^ This progress is due to the growing availability of large text corpora from the internet and the emergence of new neural network models, transformers, capable of billions of learnable parameters.^58^ Activation of these models has been shown not only to develop human-like conversational capabilities,^59^ but also to generate patterns similar to those of the human brain.^60^^,^^61^^,^^62^^,^^63^^,^^64^^,^^65^ Knowing that this mapping primarily relies on the algorithms' capacity for incremental processing, i.e., to predict future words from their nearby context,^60^ it suggests that this simple goal is sufficient for the algorithms to converge to computations that resemble those of the human brain.

Although the combinations are potentially endless, human speech utterances are often formulaic and typically also fragmentary. Nevertheless, understanding spoken language is an astonishingly effortless, immediate and surprisingly accurate process, suggesting that listeners possess specific mechanisms to deal with structurally complex and often incomplete acoustic input. There is solid evidence that listeners interpret the meaning of a spoken utterance long before and regardless of the end of the utterance.^66^^,^^67^ This is done by a brain that continuously generates models of the world in its higher cortical areas, which are then used to predict forthcoming sensory input.^68^ If errors are detected due to a mismatch between predicted and real sensory input, the model updates itself.^35^ Such predictive coding,^35^^,^^36^^,^^37^^,^^38^ that is, dealing with the constant stream of information by predicting incoming sensory information based on prior experience, is an economical solution, as it allows computational resources to be allocated to novel or surprising input.^37^

There is consensus that predictive coding is the way by which the human brain operates and that this includes attending to speech signals. Evidence is from experimental paradigms based on word or phonetic surprisal, i.e., the extent to which a word or sound pattern is expected, which results in quantifiable brain signals in functional magnetic resonance imaging,^69^^,^^70^ electroencephalography,^71^ electrocorticography,^72^ or magnetoencephalography.^73^ These real-time decisions are significantly dependent on combining current linguistic (e.g., lexical) and non-linguistic (e.g., contextual) sensory information with pre-existing expectations about intended meanings.^74^^,^^75^ In humans, meaning from a sentence is not heard from the start of the vocal production. When listening to speech, meaning gets computed incrementally, with each word adding to disambiguation.^76^^,^^77^ Some comparative studies with animals have argued that brain mechanisms for predictive perception and action have gradually evolved from simpler predictive loops present in earlier animal ancestral brains,^78^^,^^79^^,^^80^ and our study contributes to this argument. Even if a direct comparison between the olive colobus communication system and human language is tenuous at best, this primate calling system may reveal something more fundamental about primate cognition; how the primate brain operates as a predictive coder.

Some of the patterns reported before were unusual and require further reflection. First, it is relevant to know that olive colobus groups always travel with larger mixed-species primate groups, typically by following a group of Diana monkeys.^81^ When these poly-specific groups encounter a predator, several individuals will produce alarm calls, but olive colobus monkeys usually wait until other monkeys’ calling efforts have diminished.^22^ Here, it is likely that callers delay their calling because they try communicating to distant recipients, such as close relatives or temporarily absent group members. Olive colobus calls can be heard much beyond the caller’s own home range,^26^ supporting the hypothesis that alarm calls function in intergroup communication, probably as a consequence of their unusual social system. There are other (less likely) hypotheses that might account for the delayed calling, such as to provide social learning opportunities for less experienced group members,^82^ to display vigor to other group members^83^ or to communicate directly to the predator.^84^

Second, as predicted we found that the initial call position already distinguished between the most important threat, eagle attacks, and less urgent dangers. Surprisingly, however, the second position did not provide any further information gain. It was only the third call position that allowed the listener to finally determine whether a call sequence was triggered by the presence of a leopard or a less important cause, falling wood. After the third position in the sequence, we did not find any further gain of information relative to the type of danger that caused the utterance, suggesting other functions, such as ensuring the information reaches receivers despite environmental noise or interference or perhaps conveying social information or fostering recruitment of neighbors.^18^^,^^44^^,^^85^ This strategy is also common in human communication, where speech is chock-full of redundancy.^86^ It is also possible that the second position carries other information, such as revealing caller identity,^19^ or perhaps there are important acoustic variants with communicative functions.^22^

Overall, our study shows that non-human primates can produce call sequences that first distinguish urgent from less urgent dangers before specifying the referents. Although we did not test this directly, the study suggests that non-human primates possess computational abilities for incremental processing and predictive coding, suggesting a shared evolutionary ancestry of a fundamental feature of how the human brain represents reality and linguistic material.

### Limitations of the study

To confirm that listeners actually take advantage of the information available in the sequences, playback experiments will be required. These should focus on how anti-predatory responses unfold along the sequences, with specific predictions at each sequence position. Also important is that olive colobus monkeys use the same call system in non-predatory situations, the topic of a forthcoming study.

Second, although it is tempting to draw parallels to human language, the comparison is superficial at best. Human languages manage extensive meaning repertoires and operate with hierarchical structures, none of which is present in olive colobus. The observed prioritization of urgency over reference reflects fundamental principles of efficient signal design rather than complex linguistic mechanisms. Yet, there is considerable acoustic gradation within the two basic call types,^22^ suggesting that the number of possible structures may be much larger than what is reported in this study, the topic of forthcoming research.

## Resource availability

### Lead contact

Further information and requests for resources and reagents should be directed to and will be fulfilled by the lead contact, Quentin Gallot (quent.gallot@gmail.com).

### Materials availability

This study did not generate new unique reagents.

### Data and code availability

• Original acoustic and environmental data have been deposited at SWISSUbase and are publicly available as of the date of publication. DOIs are listed in the key resources table.
• All original code has been deposited at SWISSUbase and is publicly available as of the date of publication. DOIs are listed in the key resources table.
• Any additional information required to reanalyze the data reported in this paper is available from the lead contact upon request.

## Acknowledgments

We thank Landri Bele, Clémentine Bodin, and Arsene Toh Gueye for assistance in setting up the experiments, and all the other members of the Taï Monkey Project for their support: Ferdinand Ouoro Bele, Ernest Kamy Biohomy, Paterson Kalo, Noël Guy Peho, Sébastien Gbamlin, Edmond Baoue. We thank Anderson Bitty and the staff of the *Centre Suisse de Recherches Scientifiques* (CSRS) for logistic support and the *Office Ivoirien des Parcs et Réserves* (OIPR) for permission to conduct research in Taï National Park. Research was funded by the 10.13039/501100001711Swiss National Science Foundation (Project Grant 310030_185324 to K.Z.; 10.13039/501100023555NCCR Evolving Language, Agreement #51NF40_180888; and PCEFP1_186841 to S.M.).

## Author contributions

Conceptualization, Q.G. and K.Z.; methodology, Q.G., Y.T., and K.Z.; formal analysis, Q.G., Y.T., and S.M.; investigation, Q.G., C.D., and K.Z.; resources, K.Z.; data curation, Q.G.; writing – original draft, Q.G.; writing – review and editing, Q.G., K.Z., Y.T., and S.M.; visualization, Q.G. and Y.T.; supervision, K.Z. and S.M.

## Declaration of interests

The authors declare no competing interests.

## STAR★Methods

### Key resources table

**Table:** 

REAGENT or RESOURCE	SOURCE	IDENTIFIER
**Deposited data**		

Original data deposited for this study	SWISSUbase	SWISSUbase: https://doi.org/10.60544/nd6p-dk04

**Experimental models: Organisms/strains**		

Olive colobus (*Procolobus verus*)	Taï National Parc, Ivory Coast	N/A

**Software and algorithms**		

Code for model building, evaluation, and plotting	SWISSUbase	SWISSUbase: https://doi.org/10.60544/0sb0-h647
R: A language and environment for statistical computing v4.3.1	R Core Team (2023)	http://www.r-project.org/
Raven Pro 1.6.4	Raven Pro: Interactive Sound Analysis Software (2023)	https://ravensoundsoftware.com/software/raven-pro/
Audacity v2.1.0	Audacity Team (2020)	https://www.audacityteam.org/
Adobe Photoshop v23.0.0	Adobe Photoshop (2019)	https://www.adobe.com/products/photoshop.html
*igraph* R package v1.4.2	Csardi & Nepusz (2005)	https://cran.r-project.org/web/packages/igraph/index.html

**Other**		

3D printable model of an African crowned eagle (*Stephanoaetus coronatus*)	SWISSUbase	SWISSUbase: https://doi.org/10.60544/g1sx-4j76

### Experimental model and subject details

Data collection in the Taï National Park was approved by the *Office Ivoirien des Parcs et Réserves* (OIPR). Research authorization and Ethics approval has been given by the *Ministère de l’enseignement supérieur et de la Recherche Scientifique* of Ivory Coast (permit number 010/ME/SRS/DGRI). The data used in this study comprise behavioral observations, obtained using playbacks and predator visual models, and sound recordings of wild, free-ranging male and female olive colobus monkeys. The precise ages of these individuals were unknown.

### Method details

#### Study site and species

This study was conducted in the Taï National Park (Ivory Coast) in an approximately 70 km^2^ study area surrounding the *Centre de Recherche en Ecologie* (WGS84: N5° 49.9′, W7° 20.5′). Data were collected during two main field periods by three different researchers (period 1: *N* = 11 different groups, KZ, 1994–1999; period 2: *N* = 18 different groups, QG, 2021–2022; Cassandre Depriester, CD, 2022; Table 1).

Olive colobus monkeys occupy small home ranges of about 0.56 km^2^,^39^ and groups are almost always found in association with other monkey species, a well-documented anti-predator strategy.^87^ Associations are mostly with Diana monkeys,^39^^,^^88^ but also with Campbell’s monkeys, lesser spot-nosed monkeys (*Cercopithecus petaurista*), King colobus (*Colobus polykomos*), red colobus (*Procolobus badius*), sooty mangabey (*Cercocebus atys*) and, much more rarely, with putty-nosed monkeys (*Cercopithecus nictitans*).^39^

In the Taï forest, monkeys have three main predators, i.e., African crowned eagles (*Stephanoaetus coronatus*), leopards (*Panthera pardus*) and chimpanzees (*Pan troglodytes*).^89^ Other dangers include falling trees or large branches,^47^ occasional lightning strikes, snake bites and human poaching.

#### Playback and predator model experiments

The first source of information - datasets 1 & 2 (Table 1) - consisted of recordings of unhabituated mixed-species monkey groups that contained olive colobus monkeys responding to playbacks of predator vocalizations (chimpanzee, eagle, leopard) and other threats (sound of falling trees). All stimuli were played with a naturally sounding amplitude range (see Methods S1 and Table S5 for more details). The experimenters systematically searched the study area until a mixed-species group was found, typically by hearing their vocalizations. After detecting a group, we determined the group’s general behavior, species composition, location, and environmental variables related to the surroundings condition (i.e., general illumination, and local vegetation density). Due to the cryptic nature of the olive colobus monkey, identifying the caller was challenging, and determining its sex was not possible during the trial. We always tried to remain undetected by avoiding visual contact with the group and monitored their behavior from between 25–75 m for at least 15 min prior to initiating a trial. To avoid pseudo replication, different versions of each playback stimulus type (*N* = 4 eagle shrieks, *N* = 4 leopard growls, *N* = 2 chimpanzee pant-hoots, *N* = 7 falling tree sounds) were edited and each version was never played more than once to each group. We avoided retesting a group with the same stimulus more than once, and did not retest any group in an area of 1 km around the location of the experiment for at least one week (olive colobus mean home range = 0.56 km^2^, corresponding to an 850-metre diameter disc^39^). We also ensured that each stimulus was never presented more than once at the same location. The monkeys’ vocalizations and behavior were recorded up to 5 min following the start of each experiment (see Methods S1 for details of the equipment used). Most vocal behavior had ceased after 160 s, so we only considered the first 160 s of recording, following the beginning of each playback trial.

The second source of information (dataset 3, Table 1) was generated by alarm calls given in response to visual predator models. We used two types of models: a leopard model (Figure S5) consisting of an experimenter covering himself with leopard-patterned fabric mimicking the size, shape and posture of a leopard, and an eagle model consisted of a camouflaged experimenter presenting a life-size 3D printed and hand-painted crowned eagle to subjects (Figure S5). Again, groups were located, monitored and the same variables related to the surroundings were recorded before trials. To avoid pseudo replication, we presented *N* = 2 different versions of each predator randomly to monkey groups. All models were presented in motion to facilitate detection. For both models, the experimenter slowly approached the periphery of a group to target a focal individual. The model remained in sight for about 1 min after detection, before slowly moving it away and hiding behind a tree trunk. We registered behavioral and vocal responses from the monkeys for up to 3 min after model detection (see Methods S1 for a detail of equipment used) but will only present the first 160s of the recordings.

### Quantification and statistical analysis

We were interested in how information was encoded along the sequences and, specifically, if context became increasingly disambiguated over the three first calls.

To address this, we plotted two tries on all datasets (*N* = 284 elicited sequences from *N* = 119 trials), a first trie showing the full sequence length and a second trie showing only the first three positions in the sequence, using *igraph* R package v1.4.2.^90^

All stimuli triggered call sequences in the study groups, except for visual eagle models (*N* = 10 trials) and chimpanzee pant-hoots (*N* = 14 trials).

Statistical analyses were done using R software v4.3.1^91^ and plotted using R and Adobe Photoshop software v23.0.0.^92^

To measure the information gain as a function of the number of calls in the sequence, we considered two approaches. The first one is based on information theory and the second one on prediction gain.

#### Notation

The letter E stands for “eagle”, L for “leopard” and T for “falling tree”. Consider the variable Y being the nature of the disturbance, which takes the values E, L, or T. The distribution of hazards is unbalanced and bears no relation to what happens naturally. Indeed, the number of vocal sequences produced in response to eagle, falling tree and leopard were, respectively, *N*_eagle_ = 39, *N*_falling tree_ = 79, *N*_leopard_ = 166. To give equal weight to each danger type, we assigned a weight to each hazard, so the sum of the weights, multiplied by the sample size, added up to one over all the observations. For eagle, falling tree, and leopard hazards, the weights were, respectively:

$$\frac{1}{3\times N_{eagle}}=\frac{1}{117};\;\frac{1}{3\times N_{falling\;tree}}=\frac{1}{237};\;\frac{1}{3\times N_{leopard}}=\frac{1}{498}$$

Before the sequence was uttered, no position was examined, the uncertainty was total, and the probabilities were:

Pr(Y=E|root)=Pr(Y=L|root)=Pr(Y=T|root)=1/3.

The probabilities associated with the beginning of the sequence were denoted, for example, by:

Pr(A),Pr(B),Pr(AA),Pr(AB),Pr(BA),Pr(BB),...,Pr(ABA).

Finally, the probabilities of variable Y conditionally on sequences of calls were denoted, for example, by:

Pr(Y=E|A),Pr(Y=L|A),Pr(Y=T|A),...,

Pr(Y=E|ABA),Pr(Y=L|ABA),Pr(Y=T|ABA).

#### Quantifying information gain

The first approach was based on Shannon entropy.^44^ The entropy was defined as:

$$H\left(0\right)=-\sum_{y\in \left\{E,L,T\right\}}\mathrm{Pr}\left(Y=y\right)\log_{3\;}\;\mathrm{Pr}\left(Y=y\right)$$

We used a logarithm in base 3, since there were three {E,L,T} categories. Before the sequence was uttered, uncertainty was total (equal to 1), as each category had a probability of 1/3. We therefore had:

$$H\left(0\right)=-\sum_{y\in \left\{E,L,T\right\}}\frac{1}{3}\;\log_{3}\;\frac{1}{3}=-\frac{3}{3}\;\log_{3}\;\frac{1}{3}=1\text{.}$$

The lower the uncertainty, the lower the entropy. If the entropy was zero, this meant that one category had a probability of 1 and the other probabilities were equal to 0. We could then calculate the entropy conditionally on each sequence. For example:

$$H\left(A\right)=-\sum_{y\in \left\{E,L,T\right\}}\mathrm{Pr}\left(Y=y\;|\;A\right)\;\log_{3}\;\mathrm{Pr}\left(Y=y\;|\;A\right)$$

or:

$$H\left(ABA\right)=-\sum_{y\in \left\{E,L,T\right\}}\mathrm{Pr}\left(Y=y\;|\;ABA\right)\;\log_{3}\;\mathrm{Pr}\left(Y=y\;|\;ABA\right)$$

where by convention 0log0=0.

Finally, we could calculate the entropy associated with first calls of a sequence. If the length was 1, we had:

H(1)=Pr(A)H(A)+Pr(B)H(B).

If the length was two, then:

H(2)=Pr(AA)H(AA)+Pr(AB)H(AB)+Pr(BA)H(BA)+Pr(BB)H(BB),

and so on. We could therefore quantify the gain in entropy as a function of the number of calls produced in the sequence. For example:

D(0|0)=0,D(1|0)=H(0)−H(1),D(2|1)=H(1)−H(2)

and so on. The measure D(·|·) was the Kullback-Leibler divergence.^42^^,^^43^ The Kullback-Leibler divergence is a fundamental index in information theory, used here to measure the information gain given by successive calls on the origin of the hazard. Kullback-Leibler divergence is normalized to lie between 0 and 1, using a base-3 logarithm.

#### Quantifying prediction gain

Another way was to examine the gain in terms of correct predictions when predicting the most likely hazard was usually done with a confusion matrix. If no call had been uttered, then each hazard had a probability of 1/3. If we predicted one of the three danger types, we had a probability of 1/3 that we predicted correctly the nature of the disturbance and 2/3 that we were wrong. This was denoted by the probability of accuracy J(0)=0.333. In addition, if we knew that the first call was A, then the probability of accuracy, i.e., the probability that the prediction was correct, was therefore:

$$J\left(A\right)=\max_{y\in \left\{E,L,T\right\}}\;\mathrm{Pr}\left(Y=y\;|\;A\right)\text{.}$$

We could therefore calculate the probability of accuracy when the length was 1 as follows:

J(1)=Pr(A)J(A)+Pr(B)J(B).

If the length was two:

J(2)=Pr(AA)J(AA)+Pr(AB)J(AB)+Pr(BA)J(BA)+Pr(BB)J(BB),

and so on.

For each step and each index, we also established a confidence interval under the hypothesis that the call position in step t does not provide any additional information to that already available in step t-1. These confidence intervals were constructed by randomizing the stimuli within the call categories defined at step t-1. We ran 3,000,000 simulations for each index. We then calculated the quantiles of order 2.5% and 97.5% to obtain a 95% confidence interval. If the index value obtained was outside the interval, this indicated that the call position at step t provided significant additional information to that already known at step t-1.

## Supporting citations

The following reference appears in the supplemental information.^93^

## Supplemental information

Document S1. Figures S1–S5, Tables S1, S2, and S5, and Methods S1

Table S3. Conditional distribution, probability, entropy, and prediction accuracy values for each possible branch of the trie, related to Figure 2

Table S4. RGB code and coloration of each node of the full trie, related to Figure 2

Data S1. Audio recordings of 3 sequence types, related to Figure 2 File S1 is an audio recording of the “BA” sequence type, related to Figure S1; “BABABA” sequence shown here was given in response to a leopard growl. File S2 is an audio recording of the “A + BA” sequence type, related to Figure S2; “AAAAAAAAAABA” sequence shown here was given in response to eagle shrieks. File S3 is an audio recording of the “BA + A” sequence type, related to Figure S3; “BAAAAAAAA” sequence shown here was given in response to a falling tree sound.
