# Supplementary material for: A primate grammar enabling incremental processing
Source: iScience. 2025 Mar 20;28(4):112229. doi: 10.1016/j.isci.2025.112229 (PMC12003008; doi:10.1016/j.isci.2025.112229)
Supplement: Document S1. Figures S1–S5, Tables S1, S2, and S5, and Methods S1 [file mmc1.pdf]

iScience, Volume *28*

Supplemental information

A primate grammar enabling incremental processing

Quentin Gallot, Yves Tillé, Cassandre Depriester, Steven Moran, and Klaus Zuberbühler

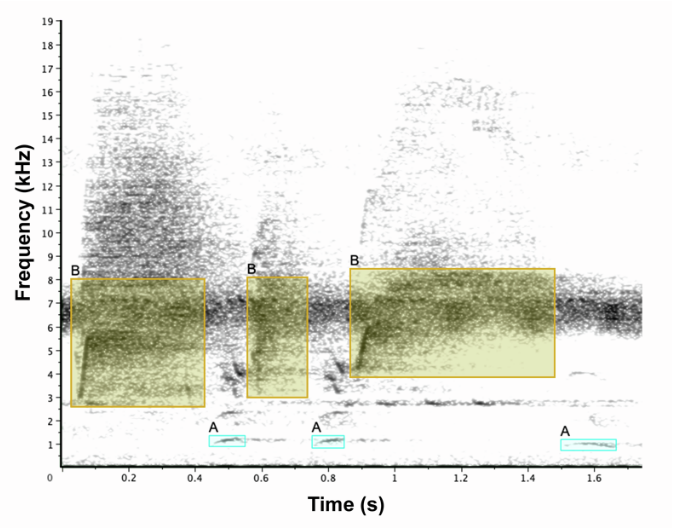

Figure S1. Spectrographic representation of the "BA" sequence type, related to Figure 2 and File S1 from Data S1. "BABABA" sequence shown here was given in response to a leopard growl. Spectrograms were extracted from Raven Pro software v1.6.4[94].

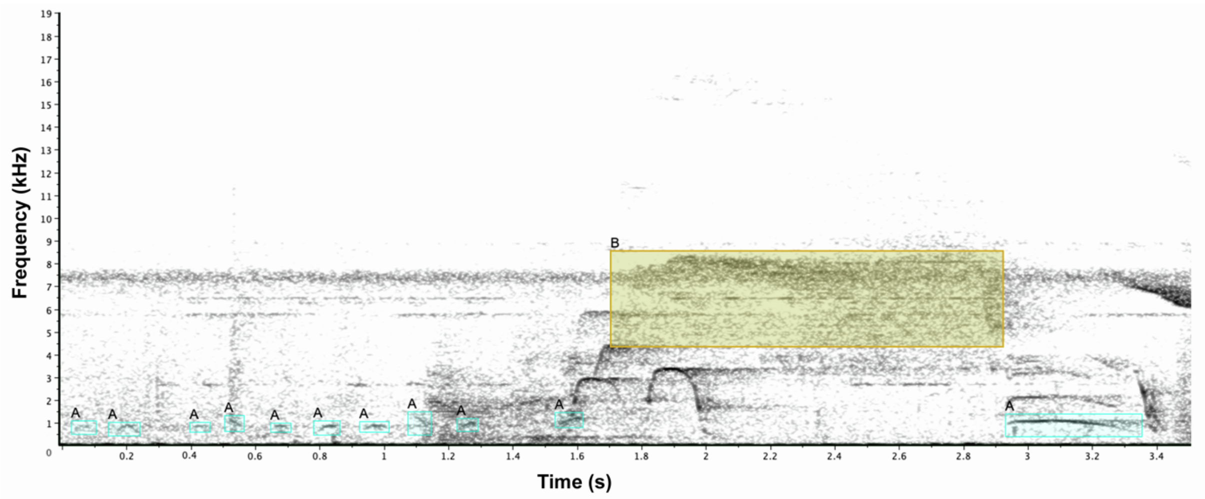

Figure S2. Spectrographic representation of the "A+BA" sequence type, related to Figure 2 and File S2 from Data S1. "AAAAAAAAAABA" sequence shown here was given in response to eagle shrieks. Spectrograms were extracted from Raven Pro software v1.6.4[94].

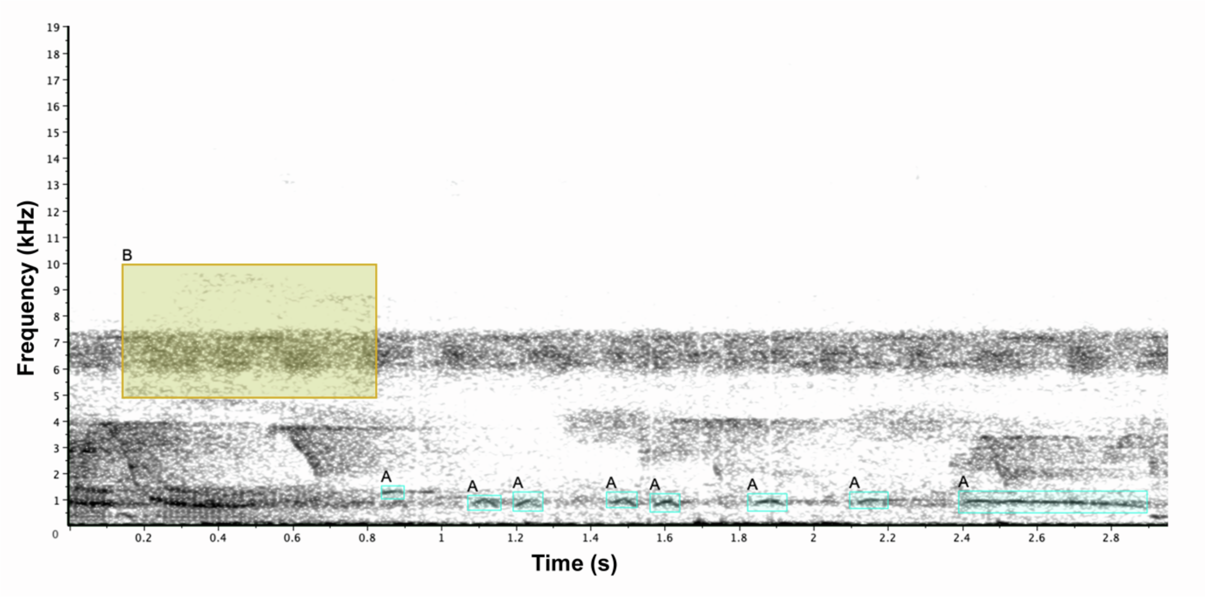

Figure S3. Spectrographic representation of the "A+BA" sequence type, related to Figure 2 and File S3 from Data S1. "BAAAAAAAA" sequence shown here was given in response to a falling tree sound. Spectrograms were extracted from Raven Pro software v1.6.4[94].

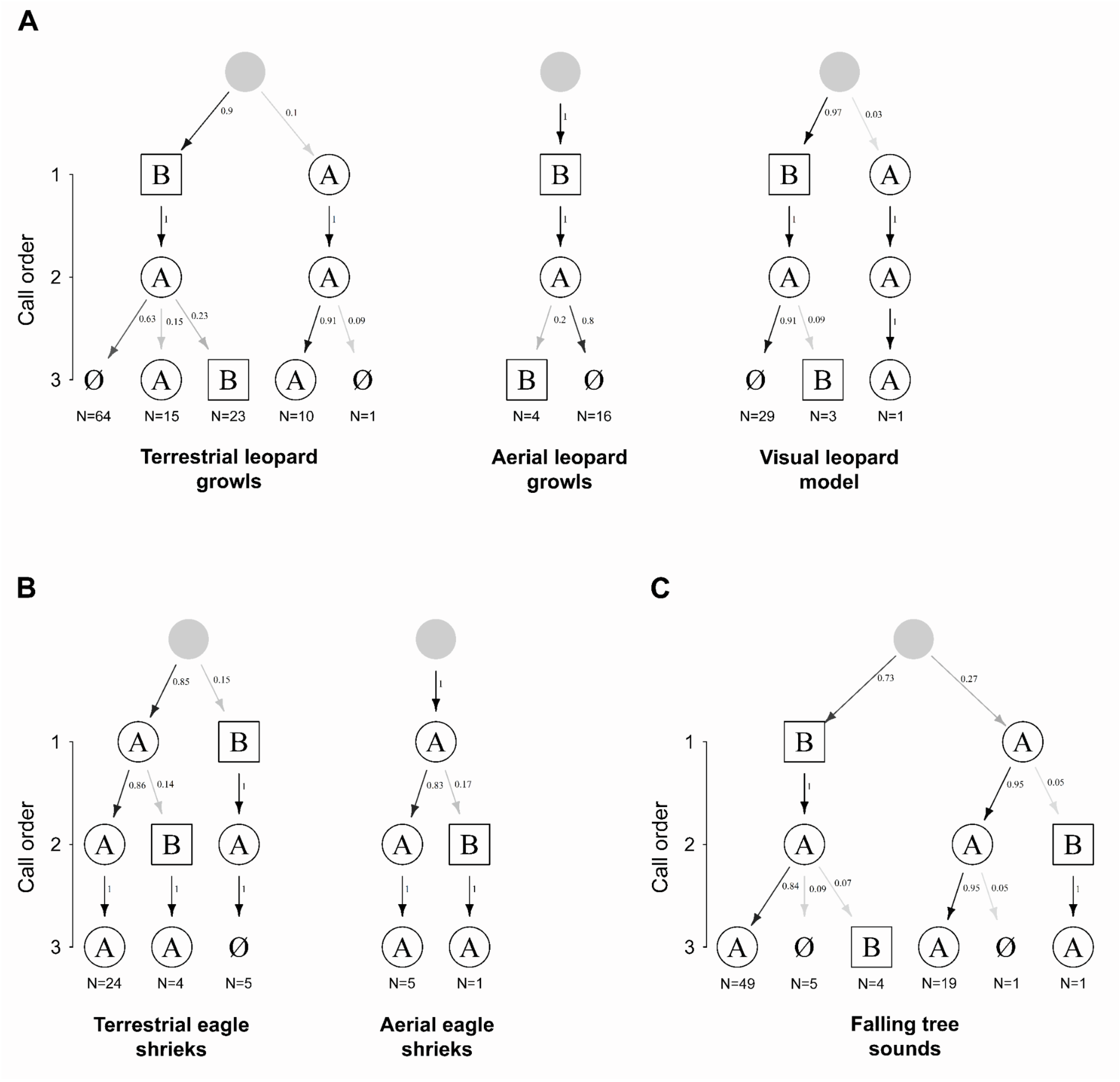

Figure S4. Tries of Olive Colobus monkey sequence's first 3 calls in response to (A) leopard, (B) eagle, and (C) falling tree danger events in relation to stimulus types, related to Figure 1. The tries were created using a different number of sequences: terrestrial leopard growls *N* = 113, aerial leopard growls *N* = 20, visual leopard model *N* = 33, terrestrial eagle shrieks *N* = 33, aerial eagle shrieks *N* = 6, and falling tree sounds *N* = 79. Each node represents a call or a position (circle = 'A', square = 'B', grey = start, 'Ø' = end). The sample size of each sequence pattern is reported at the end of each branch. The y-coordinates represent the position of the call in the sequence, the arrow direction represents the order of combination, and the arrow color grade and label represent the transition probability values between 2 nodes (1 being black and 0 being white).

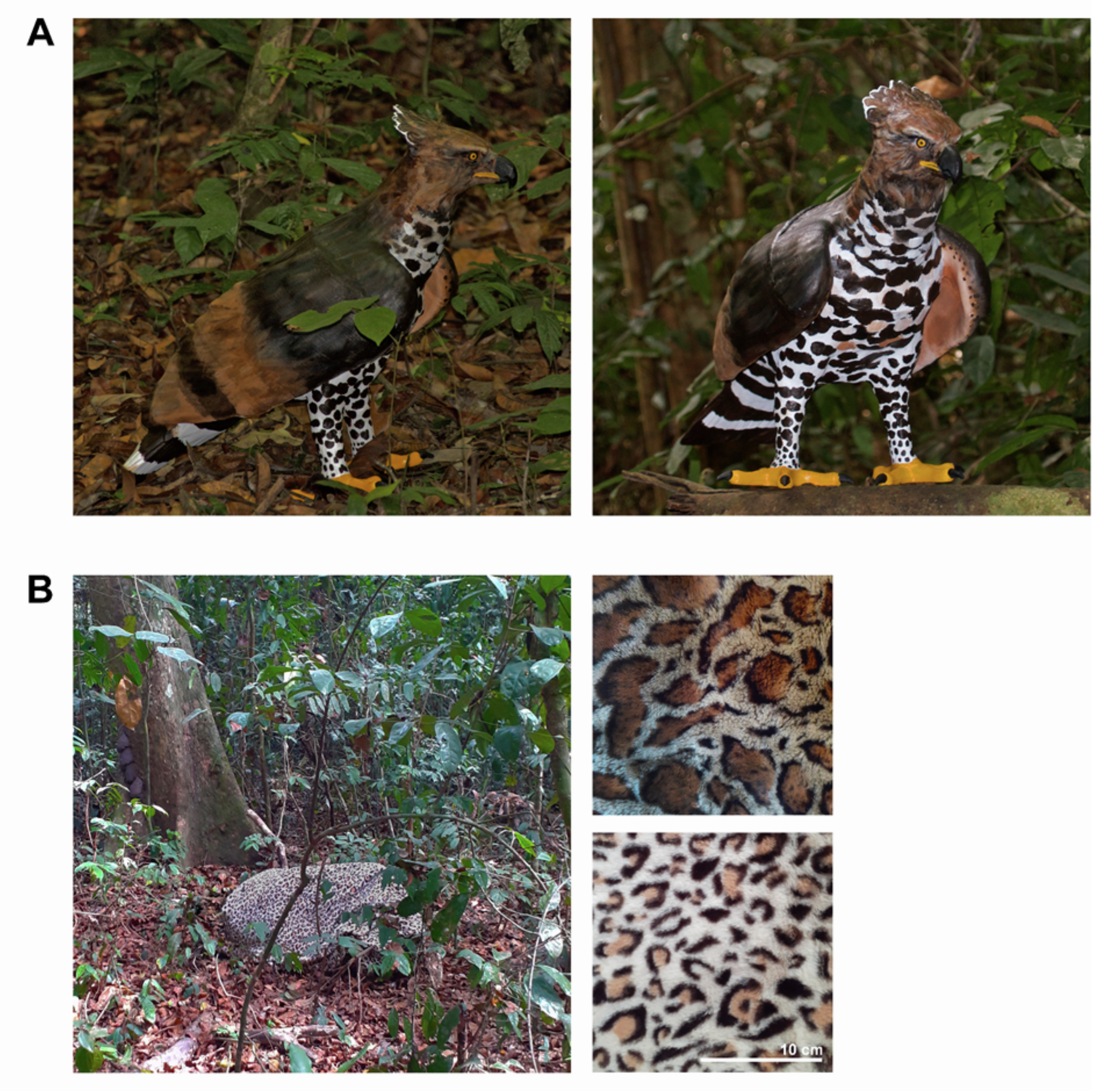

Figure S5. Lifesize animal models presented to unhabituated Olive Colobus groups, related to STAR Methods. (A) Two differently painted versions of a life-sized, anatomically-correct African crowned eagle. We 3D-printed the model using the UltiMaker Cura software v5.2.2 and the Anycubic Chiron 3D printer. (B) An experimenter on the ground with his head and body completely covered by one of two leopard fur patterned fabrics, mimicking the size, shape, and posture of a leopard.

Table S1. Probabilities of production associated with the beginning of the alarm sequence in response to three danger events (leopard, eagle, falling tree), related to Figure 1.

Call position	Sequence i	Pr(i)
0	*root*	1.00
1	*A*	0.40
	B	0.60
2	*AA*	0.36
	AB	0.05
	BA	0.60
	BB	0.00
3	*AAØ*	< 0.01
	AAA	0.35
	AAB	0.00
	ABØ	0.00
	ABA	0.05
	ABB	0.00
	BAØ	0.28
	BAA	0.24
	BAB	0.08
	BBØ	0.00
	BBA	0.00
	BBB	0.00

Table S2. Variation of the entropy H, prediction accuracy J, and Kullback-Leibler divergence D as a function of the number of calls produced in the sequence, related to Figure 1 and Figure 3. 'obs' corresponds to an index calculated with observed data, and 'sim' corresponds to an index 95% confidence interval calculated with simulated data (*N* = 3'000'000 permutations). These confidence intervals for level t were constructed by randomizing the stimuli within the call categories defined at level t-1.

Level	H_{obs}	H_{sim}	J_{obs}	J_{sim}	D_{obs}	D_{sim}
0	1.000	[1.000,1.000]	0.333	[0.333,0.333]	0.000	[0.000,0.000]
1	0.757	[0.982,1.000]	0.600	[0.338,0.398]	0.243	[0.000,0.018]
2	0.751	[0.740,0.757]	0.600	[0.600,0.608]	0.006	[0.000,0.017]
3	0.551	[0.714,0.747]	0.781	[0.600,0.627]	0.200	[0.003,0.037]
4	0.533	[0.520,0.547]	0.781	[0.781,0.795]	0.018	[0.004,0.031]
5	0.529	[0.501,0.529]	0.781	[0.781,0.794]	0.004	[0.004,0.032]
6	0.502	[0.485,0.510]	0.793	[0.787,0.804]	0.027	[0.019,0.044]
7	0.490	[0.465,0.496]	0.793	[0.793,0.804]	0.011	[0.006,0.037]
8	0.464	[0.464,0.485]	0.808	[0.795,0.808]	0.027	[0.005,0.027]
9	0.458	[0.448,0.463]	0.808	[0.808,0.816]	0.006	[0.001,0.016]
10	0.453	[0.434,0.455]	0.808	[0.808,0.816]	0.005	[0.003,0.024]
11	0.451	[0.439,0.451]	0.808	[0.808,0.812]	0.002	[0.002,0.014]
12	0.439	[0.431,0.449]	0.812	[0.808,0.816]	0.012	[0.003,0.021]
13	0.431	[0.418,0.435]	0.812	[0.812,0.818]	0.008	[0.004,0.021]
14	0.428	[0.420,0.428]	0.814	[0.814,0.816]	0.003	[0.003,0.011]
15	0.422	[0.418,0.422]	0.816	[0.816,0.818]	0.005	[0.005,0.009]
16	0.415	[0.415,0.415]	0.820	[0.820,0.820]	0.007	[0.007,0.007]
17	0.415	[0.415,0.415]	0.820	[0.820,0.820]	0.000	[0.000,0.000]
18	0.415	[0.415,0.415]	0.820	[0.820,0.820]	0.000	[0.000,0.000]
19	0.415	[0.415,0.415]	0.820	[0.820,0.820]	0.000	[0.000,0.000]
20	0.415	[0.415,0.415]	0.820	[0.820,0.820]	0.000	[0.000,0.000]
21	0.415	[0.415,0.415]	0.820	[0.820,0.820]	0.000	[0.000,0.000]
22	0.415	[0.415,0.415]	0.820	[0.820,0.820]	0.000	[0.000,0.000]
23	0.415	[0.415,0.415]	0.820	[0.820,0.820]	0.000	[0.000,0.000]

Table S5. Amplitude measurements of all sound stimuli used during playback experiments, related to STAR Methods

			At 50cm of the speaker		At 100cm of the speaker			
Stimulus category	Stimulus	Experi-menter *	Average amplitude (dB)	Maximum amplitude (dB)	Average amplitude (dB)	Maximum amplitude (dB)	Nagra speaker 'sensitivity'	Alpha speaker 'gain'
Chimpanzee pant-hoots	C(PH)1	QG	100	103.7	96.5	100.4	-21	NA
	C(PH)2	QG	101	102.5	96	101.7	-21	NA
Eagle shrieks	E1	QG	94.5	99.7	91	92.5	-12	NA
	E2	QG	96.5	98.7	92.5	93.9	-30	NA
	E3	QG	97	99.0	92	93.6	-30	NA
Leopard growls	L1	QG	91.5	101.8	90	96.1	-35	NA
	L2	QG	93.5	102.2	91	95.4	-35	NA
	L3	QG	93	102.4	88.5	90.1	-30	NA
Falling tree sounds	TREE1	QG	97	101.7	91	95.4	-40	NA
	TREE1_short	CD	93.2	101.5	89.9	97.3	NA	2.75
	TREE2	QG	96	100.4	92.5	97.1	-21	NA
	TREE2_short	CD	86.9	100.5	83	96.9	NA	3.5
	TREE4	CD	91.6	101.9	86.8	97.6	NA	4.5
	TREE5	CD	92.4	99.9	88.8	96.7	NA	3.5
	TREE6	CD	92	101.2	87.4	97.3	NA	4

*QG: Quentin Gallot / CD: Cassandre Depriester. All the stimuli used by KZ were played with a naturally sounding range with a maximum amplitude at 100 cm of the speaker between 88 and 100dB (leopard growls: 88 to 92 dB, and eagle shrieks: 92 to 100 dB).

Methods S1. Equipment and protocol details, related to STAR Methods

Dataset 1 & 2: Playback experiments

Leopard growls were purchased from the British Library of Wildlife Sounds (BBC master tape number MM35, South African Broadcasting Corporation), eagle and chimpanzee vocalizations were recorded by KZ in the study area, falling tree sounds were recorded by KZ in the study area or purchased from BBC Sound Effects library (sounds 07002293, 07058030, 07058129).

To determine if the location of the predator is encoded in the vocal response of Olive Colobus, the loudspeaker was placed at different heights in the canopy for the leopard and eagle playback trials. The speaker was placed either 'down' (i.e., 0 to 2 meters from the ground) or 'up' in the canopy (i.e., from 2 to 30 meters from the ground). In the 'up' condition, the loudspeaker was suspended from a high branch after placing a rope with a slingshot, outside the group's visual range. The speaker was always placed 'down' in the falling tree and chimpanzee playback trials.

Equipment

CD, and QG determined the location of monkey groups with a Garmin GPS RINO 655t, a Garmin GPS map 62s. KZ determined the location using a customized map of the forest. QG played back sounds from a Samsung Galaxy XCover 4 (model SM-G390F, with media sound on maximum volume) connected to a Nagra Kudelski DSM-monitor loudspeaker (frequency response 60–15,000 Hz ± 4 dB, Fi. S2-2). CD played back sounds from an iPhone 5S (iOS 12.5.4) connected to an Alpha speaker (AER, The Acoustic People: frequency range 60–18,000 Hz). KZ played back sounds from a Sony WMD6C Professional Walkman connected to a Nagra Kudelski DSM-monitor loudspeaker. To simulate events as naturally and consistently as possible, stimulus amplitude was calibrated at 1m from the speaker with sound level meters (QG: Standard ST-85C; CD: Decibel X app v9.4.0 on an iPhone 5S iOS 12.5.4; KZ: Radio Shack sound level meter 33–2050). Vocalizations were tape-recorded with a Sony WMD6C or TCM5000EV cassette recorder and a Sennheiser ME88 or ME67 directional microphone (frequency response, 40–20,000 Hz ± 2.5 dB; KZ; 1994-1999) or a Marantz solid-state recorder (PMD 661 MKII, 44.1 kHz sampling rate, 16 bits accuracy, WAV format) and a Sennheiser MKH 416 P48 directional microphone (frequency response, 40–20,000 Hz ± 2.5 dB; QG, and CD; 2021–2022). It was not possible to record data blindly because our study involved animals in the field. Prior to analysis, all recordings contained in KZ audio tapes were digitized (44.1 kHz sampling rate, 16 bits accuracy, WAV format) with Audacity software v2.1.0 software using a TASCAM CD-A500 and a Technics M280 cassette player.
